# Radiographic Thrombus within the External Jugular Vein: Report of a Rare Case and Review of the Literature

**DOI:** 10.1155/2015/807268

**Published:** 2015-10-13

**Authors:** Sayyad Yaseen Zia, Richard L. Bakst, Qiusheng Si, Mike Yao, Peter M. Som

**Affiliations:** ^1^Department of Radiation Oncology, Icahn School of Medicine at Mount Sinai Hospital, New York, NY, USA; ^2^Department of Pathology, Icahn School of Medicine at Mount Sinai Hospital, New York, NY, USA; ^3^Department of Otolaryngology, Icahn School of Medicine at Mount Sinai Hospital, New York, NY, USA; ^4^Department of Radiology, Icahn School of Medicine at Mount Sinai Hospital, New York, NY, USA

## Abstract

We are reporting a case of a 91-year-old male with a primary malignancy of the right parotid gland with radiographic thrombus extension within the right external jugular vein. He was treated with palliative radiation therapy to the right parotid mass with a marked clinical response. The rarity of this occurrence as documented in the review of the literature provides for uncertainty with regard to proper management. Radiographic evidence of thrombus in the absence of clinical manifestations, the role of anticoagulation, and the proper radiation target delineation were all challenges encountered in the care of this patient. Our case represents a rare occurrence with unique radiologic findings that has implications for management.

## 1. Introduction

We are reporting a case of a 91-year-old male with a primary malignancy of the right parotid gland with thrombus extension within the right external jugular vein. He was treated with palliative radiotherapy to the right parotid mass with a marked clinical response. Tumor thrombus within neck veins is an uncommon event and in the head and neck it has most often been reported in association with thyroid malignancies [1–6]. Thrombus associated with malignancy may manifest clinically but in our case was detected radiographically. Here we report a case of a parotid malignancy with direct intravenous thrombus extending down the right external jugular vein to the level of the subclavian vein documented with computed tomography (CT) and positron emission tomography (PET) and review the literature on this rare radiographic finding.

## 2. A Case Report

A 91-year-old male with no smoking, drinking, or prior malignancy history was initially referred to the Department of Radiation Oncology for palliative irradiation to a growing painless right parotid mass that had been present for one year.

One year prior to presenting to our clinic, the patient had noticed a right neck mass. CT of the neck demonstrated an unencapsulated, poorly defined hypodense mass measuring 2.3 × 1.5 × 2.1 cm in the superficial portion of the right parotid gland with fine needle aspiration (FNA) suggesting Warthin tumor. Further management was complicated by a stroke that left him wheelchair bound with aphasia. CT of the neck one year later noted the mass to have increased in size to 8.8 × 6 × 4 cm with no evidence of associated thrombus.

**Figure 1:**
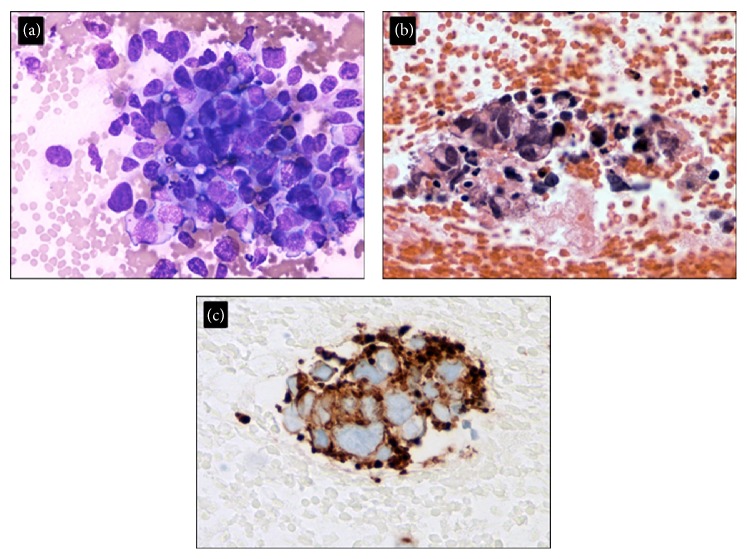
Parotid tumor pathology. Smear and cell block section from FNA biopsy showing pleomorphic malignant cells ((a) Diff-Quick stain, ×600; (b) cell block, ×600). The tumor cells are positive for Cam 5.2 ((c) ×600) and negative for AE1/AE3, p63, and CK5/6. Melan A, androgen receptor, mammaglobin, S-100, CD45, CD3, CD20, and TTF1. The cytological features with immunostaining results support a high grade carcinoma.

**Figure 2:**
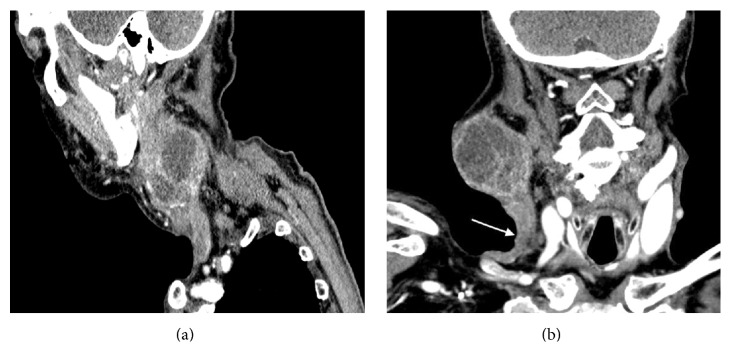
CT neck: sagittal and coronal images with contrast demonstrating parotid tumor with external jugular vein invasion with extension to the level of the right subclavian vein.

**Figure 3:**
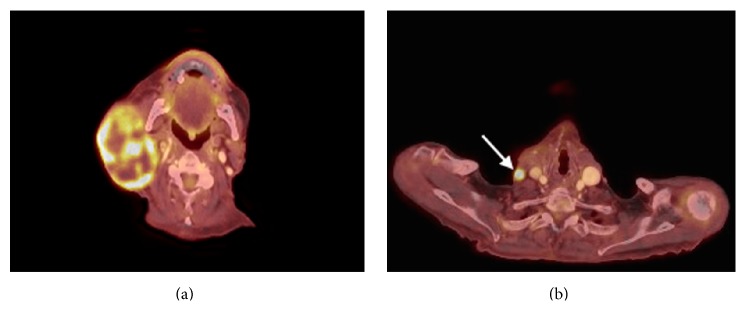
PET: axial images demonstrating FDG avidity in right parotid mass and extension to right subclavian vein.

Upon presentation at our institution, physical examination demonstrated significant enlargement in the size of the mass with no evidence of swelling, palpable veins, or tenderness in the neck. FNA of the mass showed pleomorphic malignant cells suggestive of a high grade carcinoma (Figure 1). CT of the neck showed a large mass in the right parotid gland measuring 8.5 × 5.4 × 8.3 cm with some invasion of the adjacent masseter and sternocleidomastoid muscles. There was invasion of the right external jugular vein with thrombus within the vein extending to the level of the right subclavian vein (Figures 2(a) and 2(b)). There was no gross evidence for invasion of the bone. PET demonstrated a large heterogeneous mass abutting the mandibular ramus measuring up to 8.6 cm with an SUV max of 14 involving the right parotid gland. Arising from the posterior region of this mass was a tubular structure with FDG avidity and internal thrombosis that appeared to form a collateral with the right brachiocephalic vein (Figures 3(a) and 3(b)). FNA of the tubular structure was negative for malignant cells with lymphocytes present. The patient was not started on any anticoagulation for the thrombus secondary to his recent stroke. A goal of care discussion deemed the patient to be medically inoperable due to his poor performance status, advanced age, and comorbidities. The patient was initiated and completed a course of radiation therapy to the parotid mass to a total dose of 6000 cGy with significant tumor shrinkage noted during treatment. On one month follow-up the patient experienced no acute side effects of radiation therapy with tumor shrinkage to 1/3 of presenting volume.

## 3. Discussion

Thrombus associated with malignancy can result from either tumor compression of a vein leading to stasis or direct extension of the primary tumor. Clinically this is important to distinguish when determining either the appropriate extent of surgical resection or radiation target volume delineation. Direct tumor extension into an adjacent vein from a head and neck malignancy is rare and was first reported by Kaufmann in 1879. Since then, less than 20 cases have been reported associated with thyroid cancer [3, 5, 7, 8]. The invasion of the internal jugular vein was from either direct tumor extension from the thyroid gland or extension from a metastatic node [3]. The presence of a tumor thrombus in the internal jugular vein from a thyroid cancer was first documented with CT in 1991 [4]. Since then there was a report of a renal cell carcinoma metastatic to the parotid gland with tumor thrombus in the adjacent veins. There are also reports of deep lobe parotid tumors causing compression thrombosis of the internal jugular vein and an associated report of a patient with an acute parotitis and a thrombosis of the internal jugular vein [9–11]. However, we could not identify a previous report of a primary parotid malignancy with direct tumor thrombus into an adjacent vein. In our case, the thrombus was in the external jugular vein extending down to the subclavian vein. There are other head and neck tumors that have been reported to invade or grow within the great vessels; among these are the paragangliomas [12, 13].

There are reports of increased FDG avidity in an intravenous tumor thrombus from a thyroid carcinoma [6]. However, there is also a reported increased intravenous FDG avidity in jugular vein thrombosis without a tumor thrombus [14]. Thus increased avidity alone within a vein is not conclusive of an intravenous tumor thrombus. The tumor thrombus is the intraluminal extension of the primary tumor, around which fibrin is deposited, protecting the tumor and allowing further growth into the vessel lumen [4].

Our case illustrates several interesting radiological findings that have implications for management. The first being the extensive nature of the thrombus associated with the tumor extending down the length of the right external jugular vein to the level of the right subclavian vein. Although in our case anticoagulation was not given, the decision to give anticoagulation should be carefully considered particularly with malignancy related hypercoagulable states. The long time course from initial detection to initiation of therapy adds ambiguity to the nature of the thrombus. During that time, either tumor extended into the vessel or prolonged stasis allowed blood thrombus formation.

The uncertainty of the nature of the thrombus with regard to whether it represented tumor or simple blood thrombus presents further therapeutic challenges, and the fact that it showed FDG avidity on PET adds another layer of opacity. As noted previously, there have been reports in the literature of increased avidity not being conclusive of tumor in a thrombus, as was in our case. Caution should be exercised when making treatment decisions solely based on increased FDG avidity. If doubt exists about the nature of the thrombus and the site in question is readily accessible, pathologically sampling could greatly aid in guiding the correct therapeutic intervention. In our case, FNA demonstrated lymphocytes, which were more consistent with blood thrombus than tumor extension. If a FNA is not feasible, a magnetic resonance imaging (MRI) scan may be useful in distinguishing between tumor and blood thrombus. Lastly, correct target delineation corresponding to the known tumor is critical in delivering safe and effective radiation therapy. If the thrombus had been treated as if it contained tumor, a far larger area would have been irradiated with minimal to no increase in tumor control or palliation of symptoms. Furthermore, increased amounts of normal tissue would have been irradiated leading to increased acute and late radiation toxicities. Our case represents a rare case with unique radiologic findings that has implications for management.
